# ABCC6 Mutation in Patients with Angioid Streaks

**Published:** 2006-02

**Authors:** Yoshihiro Mizutani, Tomohiro Nakayama, Satoshi Asai, Hiroyuki Shimada, Mitsuko Yuzawa

**Affiliations:** 1 *Department of Ophthalmology, Nihon University School of Medicine, Tokyo, Japan;*; 2 *Division of Receptor Biology, Advanced Medical Research Center, Nihon University School of Medicine, Tokyo, Japan;*; 3 *Division of Genetic & Genomic Research, Advanced Medical Research Center, Nihon University School of Medicine, Tokyo, Japan*

**Keywords:** ABCC6 gene, angioid streaks, choroidal neovascularization, missense mutation, pseudoxanthoma elasticum

## Abstract

Angioid streaks (AS) are hereditary eye conditions caused by breaks in the elastic layer of Bruch’s membrane. Patients with AS are also frequently affected with pseudoxanthoma elasticum (PXE). The locus of PXE has been reported to exist in chromosome 16p13.1, and the ABCC6 gene in this locus has been identified as the causal gene of PXE. In this study we investigated the association of the ABCC6 gene and AS. Elucidation of the causal gene of AS will be useful for gene diagnosis in the future. Many mutations in patients with PXE are found in exons 24 and 27 of the ABCC6 gene in previous reports. Therefore, we examined exons 24 and 27 of the ABCC6 gene using the single-strand conformation polymorphism technique. There was no mutation or polymorphism in exon 24. The base substitution of G3803A was identified in exon 27, with a change in the amino acid from CGG to CAG (R1268Q). The genotype frequencies in patients with AS were G/G 52% (23/44), G/A 32% (14/44) and A/A 16% (14/44). In control subjects, the genotype frequencies were G/G 69% (107/154), G/A 29% (44/154) and A/A 2% (3/154). Highly significant differences were observed in both genotype and allele frequencies of R1268Q between patients with AS and control subjects (*p*<0.001, *p*<0.002; chi-square test). In conclusion, the missense mutation R1268Q in the ABCC6 gene is not a specific marker of PXE, but is associated with the disease state of AS.

## INTRODUCTION AND METHODS

Angioid streaks (AS) (OMIM 607140) are hereditary eye condition caused by breaks in the elastic layer of Bruch’s membrane. However the inheritance pattern for AS has not yet been clarified. Although visual acuity does not decrease due to the presence of angioid streaks themselves, visual loss does occur due to the secondary hemorrhage and/or exudation from choroidal neovascularization (CNV) that develops through the streaks in the macula. About 70% of patients with AS experience decrease in central visual acuity by the age of 50 (1, 2). AS have been reported in many systemic disorders including pseudoxanthoma elasticum (PXE) (1), Paget disease of bone (3), sickle cell anemia (4), and Ehlers-Danlos syndrome (5). The most common disorder associated with AS is PXE. Approximately 60% of patients with AS will have PXE (1).

On the other hand, PXE is a rare disease of the connective tissue and it exhibits an autosomal recessive (OMIM 264800) or an autosomal dominant (OMIM 177850) inheritance pattern. It is characterized by progressive calcification of elastic fibers in the skin, Bruch’s membrane of the eye, and the cardiovascular system. The association of AS with PXE was first reported by Groenblad (6) and Strandberg (7) in 1929. AS are estimated to be present in about 80% of patients with PXE.

**Figure 1:**
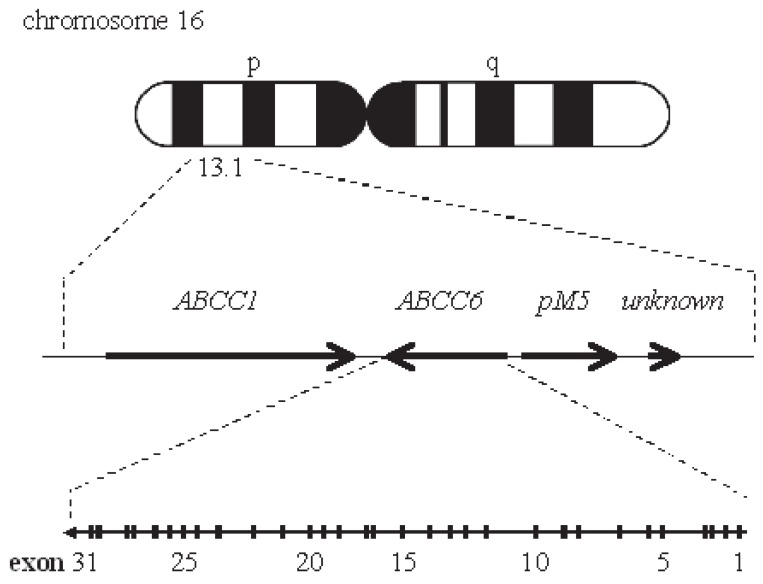
The location of the ABCC6 gene and neighboring genes in 16p13.1. Arrows indicate genes, and the direction of translation.

Previous studies using positional cloning have mapped the locus of the autosomal recessive or dominant inheritance forms of PXE to chromosome 16p13.1 (8, 9) and subsequent studies refined this locus to a 500-kb region (10, 11). The ABCC6 gene is known to be in this region, and has been identified as the causal gene of PXE (12-15) (Fig. 1). However, no report so far has examined the association between AS and the PXE causal gene ABCC6, despite the frequent association between AS and PXE. In this study we examined the possible mutations or polymorphisms in the ABCC6 gene, and investigated the association between the ABCC6 gene and AS. Elucidation of the causal gene of AS will be useful for the development of gene diagnosis and gene therapy in the future.

We enrolled 44 consecutive patients between October 2001 and April 2002 at Surugadai Hospital, Nihon University. There were 27 males (age range 49-78 years) and 17 females (age range 19-71 years) aged between 19 and 78 years with a mean ± SD of 61.2 ± 9.7 years. Except for a 19 year-old female, all other patients were older than 48 years of age. Ophthalmological examinations including measurement of visual acuity, fundus examination, and fluorescein angiography (FA) were conducted in all patients. CNV associated with AS was diagnosed based on fundus examination and FA. Dermatological examination and/or skin biopsy were conducted for the diagnosis of PXE. While a diagnosis of positive PXE was made by macroscopic dermatological examination or skin biopsy, a diagnosis of negative PXE was always based on a skin biopsy. As a result, PXE was positive in 27 patients (include a 19 years old female) and negative in 5 patients, while PXE status was unclear in the remaining 12 patients because they refused to undergo skin biopsy.

As controls, we investigated 154 healthy unrelated Japanese subjects (111 males, age range 50-81 years; and 43 females, age range 50-73 years) aged between 50 and 81 years with a mean of 57.4 ± 6.4 years. All control subjects were healthy volunteers who visited the Comprehensive Health Evaluation Center of Nihon University School of Medicine for a routine medical checkup. They had no remarkable medical history including AS documented by fundus examinations. Their best-corrected vision was 20/20 or better.

Informed consent was obtained from all subjects as per the protocol approved by the ethical committee of Nihon University. This investigation was performed according to the guidelines of the Declaration of Helsinki.

Genomic DNA was extracted from whole blood according to standard procedures (16). Data of the ABCC6 gene sequence were obtained from the published sequence of the human chromosome 16 BAC clone A-962B4 (GenBank Accession No. U91318) (17) and the exon-intron boundaries of the 31 exons in the ABCC6 gene were detected by comparison with the published cDNA sequence data (GenBank Accession No. AF076622) (18). Genomic DNA from each patient was screened for sequence variations in exon 24 and exon 27 of the ABCC6 gene by single-strand conformation polymorphism (SSCP) analysis (19) as these regions are considered to be the hot spots of mutations. Four pairs of oligonucleotide primers were designed for SSCP, and all primers were labeled with Texas Red (Table 1). Polymerase chain reaction (PCR) was performed in a 10 μl amplification reaction mixture containing 200 ng human genomic DNA, 2 pmol of each PCR primer, 2.5 mM MgCl_2_, 0.2 mM of each dNTP, and 0.5 units LA Taq polymerase (5 units/μl) (LA PCR kit Ver. 2, Takara Syuzo, Tokyo, Japan). The thermoprofile was 94°C for 3 minutes, followed by 35 cycles of 98°C for 25 seconds, 63°C for 30 seconds, 72°C for 1 minute, and a final extension at 72°C for 10 minutes. Samples were heat denatured, and electrophoresed with two different 5% polyacrylamide gels, one with and one without 5% glycerol, using an automated LASER DNA Analyzer (SQ5500E, Hitachi High-Technologies, Tokyo, Japan). Samples that exhibited an abnormal migration band for the SSCP method were reamplified, and sequenced directly after subcloning, using an automated DNA sequencer (PRISM 310 Genetic Analyzer; Applied Biosystems, Foster City, CA).

**Table 1:** Primers used for SSCP analysis

Exon	Primers

24	Ex24-1F: 5’-GGGGCTCTCTGTGCTTCTGGAAACT-3’
	Ex24-1R: 5’-ACAAAGGGGGCCTGGGTTCGGAATC-3’
24	Ex24-2F: 5’-TGAGACGTTCCAGGCCAGCACAGT-3’
	Ex24-2R: 5’-GACCTCAGGTCTCACCCTCTAAGG-3’
27	Ex27-1F: 5’-CTGAAGCTGATAGAGGTGGGCCATC-3’
	Ex27-1R: 5’-TTGAAGGACACGCCCTGCACAGCCA-3’
27	Ex27-2F: 5’-GGGACTTTGGGCTAAGATACCGACC-3’
	Ex27-2R: 5’CCTGGAGTCCTTTGGCCTAAACTCC-3’

The restriction enzyme fragment length polymorphism (RFLP) method was used in genotyping. The PCR products for exon 27 were generated using the primers 5’-CTGAAGCTGATAGAGGTGGGCCATC-3’, and 5’-TTGAAGGACACGCCCTGCACAGCCA-3’. In a reaction mixture of 20 μl, polymerase chain reaction was performed using 200 ng human genomic DNA, 4 pmol of each PCR primer, 2.5 mM MgCl_2_, 0.2 mM of each dNTP, 5 units LA Taq polymerase (Takara Syuzo, Tokyo, Japan), and 1 x LA Taq polymerase buffer. The thermoprofile was the same as for the SSCP procedure. The PCR products were digested with BstXI and electrophoresed on 1.5% agarose gels. These products were stained with ethidium bromide, and visualized under ultraviolet light. The G/G genotype exhibits a single band of 207 bp, the G/A genotype exhibits three bands of 207 bp and 134 bp, 73 bp, and the A/A genotype exhibits two bands of 134 bp and 73 bp.

Data are presented as mean ± SD. All statistical analyses were done with StatView, ver. 5.0. The chi-square test was used in all statistical analyses. *P* values less than 0.05 were considered to be significant differences.

## RESULTS AND DISCUSSION

**Figure 2:**
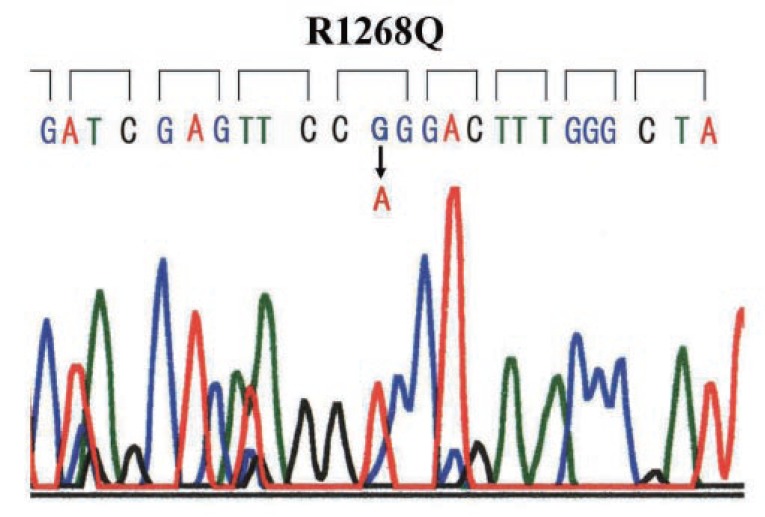
Nucleotide sequence of exon 27 of the ABCC6 gene. Arrow indicates the nucleotide constitution with a change in the amino acid (R1268Q). Nucleotide sequence indicated the A/A homozygotes.

The SSCP analysis showed no abnormal migration band in exon 24 of the ABCC6 gene. In exon 27, abnormal migration bands were detected. After direct sequencing, base substitution of G3803A was identified in exon 27. This substitution yields an amino acid change from CGG (Arg) to CAG (Gln) (R1268Q) (Fig. 2).

**Figure 3:**
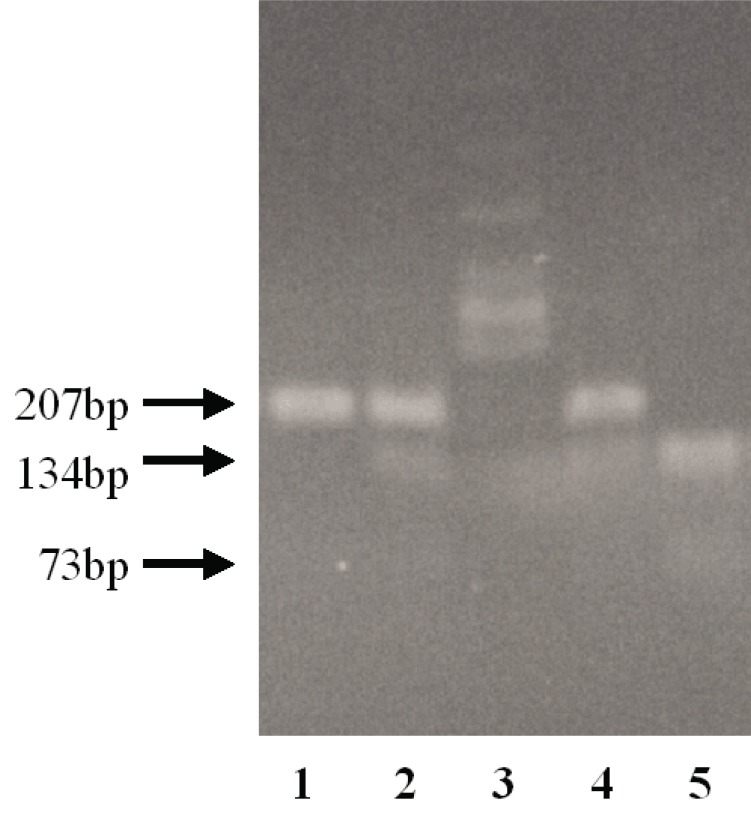
Electrophoresis of RFLP. The G/G genotype exhibits a single band of 207 bp (Lane 1). The G/A genotype exhibits three bands of 207 bp, 134 bp, and 73 bp (Lanes 2 and 4). The A/A genotype exhibits two bands of 134 bp and 73 bp (Lane 5). Lane 3 shows a DNA molecular marker (φ174 Hinc II digested). Although the band for 73bp was too weak to be detected by photography, it did not present a problem to discriminate between the genotypes.

We investigated the frequencies of the G3803A (R1268Q) genotypes by the RFLP method (Fig. 3). In the AS subjects studied, the genotype frequencies were 52% (23/44), G/A 32% (14/44) and A/A 16% (7/44). No significant difference in allele frequency was observed between patients with and without PXE (*p*=0.69) (Table 2).

**Table 2:** Comparison of the genotypes between AS and Control subjects

AS total	(n=44)	23 (52%)	14 (32%)	7 (16%)
PXE (+)	(n=27)	15	10	2
PXE (−)	(n=5)	4	0	1
not examined	(n=12)	4	4	4
Control	(n=154)	107 (69%)	44 (29%)	3 (2%)

AS = angioid streaks; PXE = pseudoxanthoma elasticum.

The genotype frequencies in the control subjects were G/G 69% (107/154), G/A 29% (44/154) and A/A 2% (3/154). These results are in agreement with the predicted Hardy-Weinberg equilibrium values (χ^2^=0.19, degrees of freedom [df]=1, *p*=0.9). A statistically significant difference in genotype frequency was observed between the patients with AS (n=44) and control subjects (n=154) (*p*<0.001) (Table 2). However, there was no statistically significant difference in the genotype frequency between the AS patients with PXE (n=27) and control subjects (n=154) (*p*=0.16) (Table 2). The frequency of the A/A genotype was significantly higher in patients with AS compared with control subjects (*p*<0.0002). A significant difference in frequency of allele A was also observed between patients with AS and control subjects (*p*<0.002).

Previous reports have documented that approximately 80% of patients with AS concurrently have PXE (1, 2). Recently, the ABCC6 gene has been identified as one of the causal genes of PXE (12-15). However, the association between AS and the ABCC6 gene has not been determined. This report is the first to study the relationship between AS and the ABCC6 gene.

The ABCC6 gene contains 31 exons. In this study, we investigated whether genetic variants exist in exon 24 and exon 27. This is based on previous reports that many mutations in patients with PXE are found in these two exons (12-15). These regions are thus considered to be the hot spots of genetic variation. On the other hand, Le Saux *et al* (20) reported that many genetic variants exist in exon 24 and exon 28. Although we did not investigate exon 28 in the present study, this exon will be examined in the future. In this study, SSCP analysis showed no abnormal migration band in exon 24. Therefore, we conclude that there is no mutation or polymorphism in exon 24 of the ABCC6 gene in patients with AS. However, some previous studies reported that R1141X mutation in exon 24 was the most frequent mutation in PXE (20, 21). This discrepancy may be due to racial difference. On the other hand, we detected a nucleotide substitution of G to A at position 3803 (G3803A) in exon 27 in patients with AS. This nucleotide substitution results in a substitution of the amino acid arginine (CGG) to glutamine (CAG) (R1268Q). The association of R1268Q with PXE has been reported, but opinions regarding this relationship remain controversial. Ringpfeil *et al* (10) reported that R1268Q was not found in control subjects, and concluded that it represented a mutation and not a polymorphism in patients with PXE. However, in their study, the number of control subjects was relatively small, consisting of only 50 unrelated, unaffected individuals. On the other hand, other studies have reported that R1268Q was a polymorphism, and not a mutation (20, 21). However, in all of the previous studies mentioned, there was no information as to whether the patients with PXE also had AS.

Germain *et al* (22) determined the frequency of R1268Q in 62 healthy Caucasian volunteers, and reported the genotype frequencies in their control subjects as G/G 66%, G/A 29% and A/A 5%. They detected no differences in genotype frequency between the control subjects and patients with PXE, and concluded that R1268Q was a harmless polymorphism. The genotype frequency of R1268Q in Caucasians is very similar to that in healthy Japanese in the present study (Table 2). There was no significant difference in genotype frequency between our Japanese controls and the reported Caucasian volunteers (*p*=0.50), suggesting that there is no racial difference in the frequency of R1268Q.

In the present study, we found significant differences both in genotype frequency and allele frequency of R1268Q between patients with AS and the controls. There was no statistically significant difference in the genotype frequency between the AS patients with PXE and control subjects. These results suggest that R1268Q may represent a genetic marker for AS rather than PXE. Germain *et al* (22) described R1268Q as a nonfunctional substitution in case control studies of patients with PXE. However, R1268Q seems to have etiological significance in patients with AS in the present study. Therefore, detection of R1268Q warrants not only examination for the skin disease PXE, but also investigations of other systemic symptoms including AS and cardiovascular system involvement.

In patients who develop AS, the streaks are generally regarded to be absent at birth (23). If this is true, then genetic diagnosis using AS-associated genes may be useful in predicting later onset or future prognosis. This information will also be useful in the development of gene therapy for the future.

Histologically, PXE is characterized by findings of elastic fibers and calcification (24) while AS is marked by basophilia as well as calcification and thinning of the retinal pigment epithelium at the ruptured site of Bruch’s membrane (1). So far, the exact function of the ABCC6 gene and its transcribed peptide remains unknown. According to a recent study, over-expression of ABCC6 mRNA was found in the liver and kidney tissues (18). However, abnormalities in these organs have not been reported in patients with AS or PXE. Uitto *et al* (25) have suggested that the calcification observed in PXE may be a secondary change. If both AS and PXE are caused by variation of the ABCC6 gene, then it may be possible to speculate that calcification of elastic fibers in Bruch’s membrane is probably a secondary change directly tied to AS.

In summary, we screened exons 24 and 27 of the ABCC6 gene in patients with AS. A single nucleotide substitution was found in exon 27, which resulted in the substitution of an amino acid (R1268Q). Significant differences in genotype and allele frequencies of R1268Q were observed between patients with AS and control subjects. However, no significant difference in allele frequency of R1268Q was found between patients with AS with and without PXE. These findings indicate that R1268Q is not a specific marker of PXE, but is a missense mutation associated with the disease state of AS. Abnormalities in the ABCC6 gene not only cause PXE but are also associated with the onset of AS.
